# Effects of a Protein-Rich, Low-Glycaemic Meal Replacement on Changes in Dietary Intake and Body Weight Following a Weight-Management Intervention—The ACOORH Trial

**DOI:** 10.3390/nu13020376

**Published:** 2021-01-26

**Authors:** Martin Röhling, Andrea Stensitzky, Camila L. P. Oliveira, Andrea Beck, Klaus Michael Braumann, Martin Halle, Dagmar Führer-Sakel, Kerstin Kempf, David McCarthy, Hans Georg Predel, Isabelle Schenkenberger, Hermann Toplak, Aloys Berg

**Affiliations:** 1 West-German Centre of Diabetes and Health, Düsseldorf Catholic Hospital Group, 40591 Düsseldorf, Germany; kerstin.kempf@wdgz.de; 2 Olympic Training Center Freiburg-Black Forest, 79117 Freiburg, Germany; ernaehrung@andrea-stensitzky.de; 3 Human Nutrition Research Unit, Department of Agricultural, Food & Nutritional Science, University of Alberta, Edmonton, AB T6G 2E1, Canada; camila@ualberta.ca; 4 Department of Medicine, Division of Endocrinology and Diabetology, Medical University of Graz, 8010 Graz, Austria; andrea.beck@medunigraz.at (A.B.); hermann.toplak@medunigraz.at (H.T.); 5 Department of Sports and Movement Medicine, Faculty of Psychology and Human Movement Sciences, University of Hamburg, 20148 Hamburg, Germany; braumann@uni-hamburg.de; 6 DZHK (German Centre for Cardiovascular Research), Partner Site Munich Heart Alliance, Munich, Germany; martin.halle@mri.tum.de; 7 Department of Prevention, Rehabilitation and Sports Medicine, Klinikum Rechts der Isar, Technical University of Munich (TUM), 80992 Munich, Germany; 8 Department of Endocrinology, Diabetes and Metabolism and Division of Laboratory Research, University Hospital Essen, University Duisburg-Essen, 45122 Essen, Germany; dagmar.fuehrer-sakel@uk-essen.de; 9 Public Health Nutrition Research Group, London Metropolitan University, London N7 8DB, UK; d.mccarthy@londonmet.ac.uk; 10 Institute of Cardiovascular Research and Sports Medicine, German Sport University Cologne, 50933 Cologne, Germany; predel@dshs-koeln.de; 11 KARDIOS, Cardioligists in Berlin, 10787 Berlin, Germany; schenkenberger@klinische-forschung-berlin.de; 12 Faculty of Medicine, University of Freiburg, 79117 Freiburg, Germany; aloys.berg@klinikum.uni-freiburg.de

**Keywords:** protein-rich diet, meal replacement, nutritional reports, weight loss

## Abstract

Although meal replacement can lead to weight reduction, there is uncertainty whether this dietary approach implemented into a lifestyle programme can improve long-term dietary intake. In this subanalysis of the *Almased Concept against Overweight and Obesity and Related Health Risk* (ACOORH) study (*n* = 463), participants with metabolic risk factors were randomly assigned to either a meal replacement-based lifestyle intervention group (INT) or a lifestyle intervention control group (CON). This subanalysis relies only on data of participants (*n* = 119) who returned correctly completed dietary records at baseline, and after 12 and 52 weeks. Both groups were not matched for nutrient composition at baseline. These data were further stratified by sex and also associated with weight change. INT showed a higher increase in protein intake related to the daily energy intake after 12 weeks (+6.37% [4.69; 8.04] vs. +2.48% [0.73; 4.23], *p* < 0.001) of intervention compared to CON. Fat and carbohydrate intake related to the daily energy intake were more strongly reduced in the INT compared to CON (both *p* < 0.01). After sex stratification, particularly INT-women increased their total protein intake after 12 (INT: +12.7 g vs. CON: −5.1 g, *p* = 0.021) and 52 weeks (INT: +5.7 g vs. CON: −16.4 g, *p* = 0.002) compared to CON. Protein intake was negatively associated with weight change (r = −0.421; *p* < 0.001) after 12 weeks. The results indicate that a protein-rich dietary strategy with a meal replacement can improve long-term nutritional intake, and was associated with weight loss.

## 1. Introduction

Lifestyle interventions comprising of exercise and healthy eating have been shown to result in clinically relevant effects regarding body composition and metabolic risk factors [1]. However, long-term adherence to these measures remains low overall [2]. In this context, meal replacement therapies with partial [3,4] or complete replacements, also known as very low energy diets [5], have been shown to be effective and appropriate for patients with obesity and related comorbidities. This therapy approach leads to improved markers of cardiometabolic risk factors, as recently documented by the ACOORH study group [6,7]. Furthermore, considering the benefits of meal replacements for the treatment of obesity and its comorbidities [3,5,6,7], this dietary strategy has been officially included as a treatment option for type 2 diabetes [8] as well as overweight and obesity [9]. Moreover, meal replacement strategies appear to be a convenient lifestyle solution (e.g., can be individually composed and used for “to-go” meals) for patients to use, which has been shown to drive greater results and at least comparable compliance compared to conventional lifestyle approaches [10,11]. Especially in regard to type 2 diabetes, there is a wealth of data showing clinically relevant effects in terms of remission rates [5] as well as weight reduction and improvement of glucose and insulin levels [1,2,4,12,13,14]. Despite these findings, there remains a general controversy about weight maintenance and the long-term effectiveness of weight management programmes [15]. However, recent findings from the 2-years follow-up of the DiRECT-study indicate that structured weight management programs with incorporated formula diets can lead to long-term benefits regarding weight maintenance and medication reduction [16,17].

Aside from the meal replacement approach, there are indications that a protein-rich diet also contributes to weight loss and maintenance [18,19]. For example, the landmark study of Larsen et al. particularly demonstrated that a modest increase in the protein content together with a modest reduction in the glycaemic index of the diet can lead to an improvement in and maintenance of weight loss [20]. However, there remains a lack of individual long-term data from dietary records of randomized controlled interventions with meal replacement showing that meal replacement strategies are able not only to lead to a reduced short-term energy intake but also to improve long-term nutritional intake with an accompanied weight reduction. Therefore, the present subanalysis of the ACOORH trial investigates the influence of a weight management invention in combination with a protein-rich and low-glycaemic meal replacement on changes in dietary intake and accompanied weight changes in participants with overweight or obesity and accompanied metabolic risk factors.

## 2. Materials and Methods

### 2.1. Study Design and Participants

**Figure 1:**
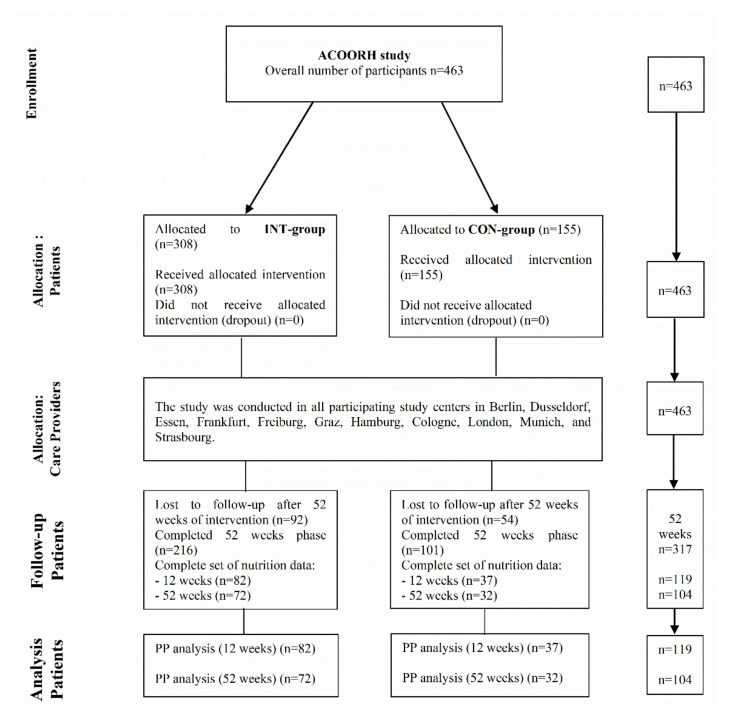
Study flow chart. CON-group, lifestyle intervention control group; INT-group, meal replacement-based lifestyle intervention group; PP, per-protocol analysis.

This subanalysis was part of the international, multicentre randomized controlled ACOORH trial (*n* = 463) [6] in participants with overweight and obesity (BMI 27–35 kg/m^2^) and accompanied metabolic risk factors. The present study was a behavioural study implementing health education and lifestyle interventions by dietary intervention and physical activity. Eligible participants were randomly assigned with a 2:1 allocation ratio to either a meal replacement-based lifestyle intervention group (INT) or a lifestyle intervention control group (CON). The lifestyle intervention was characterised by an initial intense 12-week intervention phase. This period was followed by a moderately intense intervention phase until week 26 and a further follow-up phase after 52 weeks. In contrast to the total ACOORH sample, in this subanalysis, only data from subjects (*n* = 119) who completed all assessments at baseline, weeks 12, and 52 were included (Figure 1). Participating centres and duration of recruitment was described in detail elsewhere [6,7]. The ACOORH trial was executed in accordance with the ethical standards laid down in the 1964 Declaration of Helsinki and its later amendments, and the research protocol was approved by different ethics committees in each country. The study was registered at drks.de under the number DRKS00006811. All participating individuals gave written informed consent before entering the study. Inclusion and exclusion criteria were described in detail elsewhere [7]. Participants visited the study centres at baseline as well as after 12, 26, and 52 weeks of follow-up. Detailed information of the study design and visits can be found elsewhere [6].

### 2.2. Intervention and Monitoring

Participants in both groups (CON, INT) received quarterly guideline booklets containing information on general healthy eating advice (limit sugar consumption; eat 3 meals/day; give preference to whole-grain foods, fruits and vegetables; limit fat and alcohol intake). In addition, participants were given advice on how to increase their daily levels of physical activity and were provided with telemonitoring devices (pedometers and scales; detailed information can be found elsewhere [7]) automatically transferring recorded data into a personalized online portal. Moreover, individuals of both groups were advised to complete a 4-day unweighed food record (2x weekend days and 2x weekdays) at baseline, as well as at the 12- and 52-weeks follow-up. The evaluation of the food records was carried out using the EBISpro nutrition system (Stuttgart, Germany) [21]. In addition, participants were instructed to record their leisure time physical activity (LTPA) via a validated questionnaire [22] at baseline, as well as at the 12- and 52-weeks follow-up.

Additionally, participants assigned to the INT group were asked to consume a meal replacement (a commercially available high-protein, soy-yogurt-honey product (Almased-Vitalkost^®^; Almased-Wellness-GmbH, Bienenbüttel, Germany)) during the first 26-week intervention phase (which has already been described in detail elsewhere [6]), and received an accompanying instruction manual. The meal replacement was characterised by a protein content of 53.3% (83% soy-protein-isolate and 17% skimmed milk yoghurt) and a very low glycaemic index (GI = 27), equivalent to 1507 kJ (360 kcal) energy per 100 g powder [23,24]. The accompanying manual provided information about meal replacement preparation as well as general facts about low-carbohydrate meals. However, this information (particularly about low-carbohydrate nutrition) was also part of the study visits and consultations, as well as the content of the quarterly guideline booklets for both groups. Individuals of the INT group were advised to note down the amount of meal replacement consumed, the number of meals replaced, and their current weight and waist circumference into their personal journal. After the 26-weeks follow-up, INT group participants were advised to manage their weight reduction by individual lifestyle changes but not encouraged to further replace their meals continuously until week 52. However, participants were allowed to replace meals when their weight reduction was compromised by events such as celebrations or vacations. At each study visit, nurses and dietitians reviewed the quarterly guideline booklets and provided dietary education and lifestyle counselling to the participants in both groups.

### 2.3. Statistical Analyses

The method as well as the assumptions of the sample size calculation for the ACOORH study have been described in detail elsewhere [7]. Data of the current subanalysis were presented as the means and standard deviations (mean ± SD), means and 95% confidence intervals (mean [95% CI]), or percentages, as appropriate. As applied in previous ACOORH publications [6,7], non-parametric data were analysed with Mann–Whitney U, Wilcoxon, or Friedman tests and parametric data with Student’s *t*-test, paired *t*-test, or analysis of variance with repeated measures to determine any differences between and within groups following the intervention. Differences in changes after 12 as well as 52 weeks between both groups were analysed using ANCOVAs adjusting for baseline values. In addition, linear regression analyses were performed to examine the associations of dietary intake and weight change after 12 and 52 weeks of intervention. Dichotomous variables, as well as frequencies, were compared by Fisher’s exact test. A per-protocol approach (completer analysis including only participants with a complete set of data) was applied in the present study. All statistical tests were two-sided, and the level of significance was set at α = 0.05. All analyses were performed using the SAS System^®^, version 9.4, under the Windows operating system. The statistical analysis was performed by an independent and external institute not involved in the study execution (ACOMED statistik^®^, Leipzig, Germany).

## 3. Results

From the initial cohort (*n* = 463), 68% (317/463) of the participants completed the study. Furthermore, posteriori performed data evaluation revealed that 38% (119/317) and 33% (104/317) of the study completers provided correctly completed dietary records after 12 and 52 weeks of intervention. The anthropometrical, clinical and dietary characteristics of the study participants are illustrated in Table 1. Baseline characteristics of the group with incomplete dietary records showed no differences compared to the group with correctly filled out food diaries (Supplementary Materials Table S1). INT (*n* = 82) or CON (*n* = 37) completers with correctly filled out dietary records were not significantly different regarding their anthropometrical and clinical characteristics, as well as dietary intake even when stratified by sex (all *p* > 0.05).

Table 2 shows the complete case analyses for the intra- and intergroup changes in the INT and CON subgroups after 12 and 52 weeks compared to baseline for participants with correctly completed food records. Overall, the INT group showed a significantly higher increase in protein intake (in relation to the daily energy intake) after 12 (+6.37% [95% CI: 4.69; 8.04] vs. +2.48% [95% CI: 0.73; 4.23], *p* < 0.001) and 52 weeks (+2.86% [95% CI: 1.40; 4.32] vs. +1.45% [0.04; 2.86], *p* = 0.052 (borderline)) of intervention, compared to the CON group. This trend was underlined by a covariance analysis with a repeated measurement analysis from baseline over 12 weeks until week 52 (*p* < 0.001).

Fat intake (−3.08% [95% CI: −4.82; −1.34] vs. +1.08% [95% CI: −1.23; 3.39], *p* = 0.006) as well as carbohydrate intake (−3.22% [95% CCI: −4.82; −1.63] vs. −3.27% [−5.52; −1.02], *p* = 0.008) in relation to the daily energy intake were significantly stronger reduced in the INT group compared to CON after 12 weeks of intervention but lost significance after 52 weeks of follow-up. Intake of component variables of carbohydrates (i.e., glucose, fructose) was also significantly more reduced in the INT group in comparison to CON (all *p* < 0.001).

After stratifying by sex, female participants of the INT group significantly increased their total protein intake after 12 (INT: +12.7 g vs. CON: −5.1 g, *p* = 0.021) and 52 weeks (INT: +5.7 g vs. CON: −16.4 g, *p* = 0.002) compared to female CON participants (Supplementary Materials Table S2).

**Figure 2:**
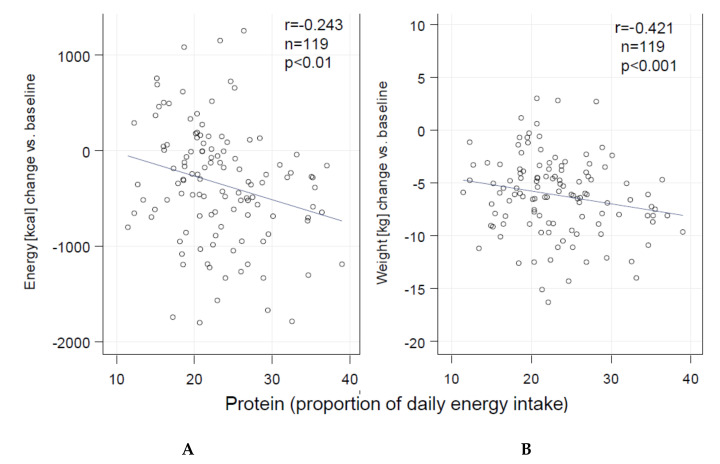
Association of protein intake (% of daily energy intake) of the whole cohort (INT + CON) (change from baseline to 12 weeks of intervention) with (**A**) energy and (**B**) weight change.

In contrast to fat and carbohydrate intake (both r= 0.373; *p* < 0.01), univariate linear regression analyses revealed that protein intake was inversely associated with both weight change (r= −0.421) and energy intake (r= −0.243) (Figure 2) after 12 weeks in the whole cohort (INT + CON) (both *p* < 0.01). These associations lost significance after 52 weeks of follow-up.

Neither FM nor FFM was associated with protein, carbohydrate or fat intake in both groups at any study visit. LTPA significantly increased within the first 12 weeks in both groups but showed no significant difference in the intergroup analysis at any time point and did also not associate with weight change or dietary intake.

## 4. Discussion

In the first 12 weeks of the present study, meal replacement effectively led to a reduced daily energy intake by limiting fat and carbohydrate consumption. However, protein intake was not affected by a reduction in energy intake. Indeed, although total energy intake was reduced (particularly in the first 12 weeks), protein intake, both absolute as well as relative to the daily energy intake, increased. Univariate regression analyses showed that this protein increase was inversely associated with weight change in the whole cohort after 12 weeks of intervention. These findings, therefore, indicate that it is possible to modify the dietary intake of participants with a high metabolic risk profile using a meal-replacement, in order to achieve a recommended protein intake for optimal health outcome, whilst at the same time being in negative energy imbalance [25,26]. The results also showed that a lifestyle intervention comprised of a meal replacement can be more effective than conventional dietary advice to increase protein intake. These results are in line with other comparable studies demonstrating that a lifestyle intervention with an accompanying meal replacement strategy can lead to weight loss as well as an increase in protein intake in older adults with obesity after 6 months of intervention [27] or to an improved overall dietary adequacy after 12 months in middle-aged women [28].

Based on the experience of the DIOGenes project [20], there is emerging scientific evidence that a diet with moderately high protein content and possibly a low glycaemic index is a precondition for weight-loss maintenance. This dietary approach has been confirmed via a wealth of data obtained from randomized clinical trials [11,18,19,29,30,31,32,33]. The findings support the recommendation for a high-protein or protein-supplemented diet for different population groups to maintain fat-free mass as well as improve body composition and metabolic biomarkers [25,34,35]. This may be of importance for many individuals, including not only participants in weight-loss programmes, but also for older adults and for normal-weight subjects and athletes who do not consume optimal dietary protein levels on a daily basis. Furthermore, especially postmenopausal women can benefit from meal replacement regimes as these products are composed of ingredients such as calcium and vitamin D supporting bone health and supplementation of necessary minerals [36,37]. However, pre-treatment macronutrient intake does not seem to correlate with weight outcomes following a 1-year lifestyle intervention [38].

Although weight change was beneficially associated with protein intake, neither FM nor FFM was associated with protein, carbohydrate or fat intake in both groups at any time point. These findings are confirmative to the current literature showing a reduction of FM and FFM [6] as a typical result of a lifestyle intervention weight loss program [39].

Besides possible beneficial effects due to the composition of the meal replacement or the change in nutritional intake, there could also be an influence of the meal replacement regime on the nutrition behaviour. This behaviour change is maybe caused as a part of a strategy to compensate for overeating and maintain dietary goals [40]. However, after the initial 26-weeks intervention phase, INT group participants were advised to manage their weight reduction by individual lifestyle changes but were not encouraged to further replace meals continuously until week 52. Participants were allowed to replace meals when their weight reduction was compromised by events such as celebrations or vacations though.

In addition, the specific mechanism(s) of action of the soy-yoghurt-honey meal-replacement product used in the present study, as well as its biological compounds such as isoflavones, soy-proteins, bio-active peptides and honey oligosaccharides, have to be considered for their influence on dietary behaviour, especially in terms of appetite regulation and energy intake [23,41,42,43,44,45].

The strengths of the present study comprise a relatively large number of participants with a detailed analysis of their dietary intakes. Furthermore, all diet diaries were evaluated by a single academic nutritionist, thus eliminating inter-assessor variations and errors.

There are, however, limitations in the present study that should be considered. In addition to the fundamental limitation of self-reported dietary records [46,47], an additional disadvantage of the present subanalysis is that the diet diaries were available in the intended form for only 119 of the 463 study participants. Besides missing data due to dropouts, the primary reasons for the loss of dietary intake data were uncompleted, incorrect, or not standardised reports. However, baseline characteristics of the completer group with correctly filled diet diaries were not different compared to the whole ACOORH cohort and, therefore, this subgroup can be assumed to be a representative sample. Moreover, based on the extent of missing data, an intention-to-treat approach was not possible to apply.

The primary intention of the intervention was to investigate the short-term (12 weeks) and long-term (52 weeks) effects of this lifestyle strategy approach on dietary intake. Thus, we did not collect dietary data after 26 weeks. Furthermore, the CON-group did not receive a control or energy-adjusted product. Moreover, the higher completer rate in the INT group compared to CON can be possibly explained by the higher success of the INT group.

To prevent overestimating of the beneficial effects of protein intake in this study, it must be taken into account that only less than 6% of the energy intake change was explained by the increase of daily protein intake in the whole group. However, weight change showed a significant (*p* < 0.001) and relevant (R^2^ ≈ 0.18) association with the daily protein intake. Therefore, the macronutrient composition has a minor but significant effect in this intervention program and indicates that macronutrient composition can be a contributing factor in a lifestyle intervention.

## 5. Conclusions

The present results of this subanalysis from the ACOORH weight management study indicate that a protein-rich and low-glycaemic meal replacement incorporated into a lifestyle intervention can improve dietary intake by increasing protein intake and decreasing fat and carbohydrate intake and can lead to successful weight loss.

## Figures and Tables

**Table 1:** Baseline characteristics of participants with correctly filled out dietary records for INT and CON stratified by sex.

Anthropometrical and Clinical Parameters	INT-Group (*n* = 82) [Men (*n* = 32) Women (*n* = 50)]	CON-Group (*n* = 37) [Men (*n* = 13) Women (*n* = 24)]
Sex (%)	39.0	35.1
Age (years)	52 ± 8	52 ± 8
Weight (kg)	92.8 ± 10.1	92.2 ± 10.4
BMI (kg/m^2^)	31.6 ± 2.4	30.8 ± 2.4
WC (cm)	107 ± 7	106 ± 8
WHR	0.97 ± 0.06	0.96 ± 0.06
FM (kg)	36.0 ± 6.0	36.3 ± 6.1
FFM (kg)	57.0 ± 5.5	55.7 ± 6.6
HbA_1c_ (%)	5.49 ± 0.33	5.45 ± 0.29
FBG (mg/dl)	92 ± 10	92 ± 11
SBP (mmHg)	135 ± 15	134 ± 17
DBP (mmHg)	92 ± 11	91 ± 8
Total cholesterol (mg/dl)	213 ± 36	218 ± 51
HDL-C (mg/dl)	53 ± 14	55 ± 10
LDL-C (mg/dl)	137 ± 31	136 ± 43
Triglycerides (mg/dl)	140 ± 73	164 ± 73

Shown are means ± standard deviations, or percentages. BMI, body mass index; DBP, diastolic blood pressure; FBI, fasting blood insulin; FBG, fasting blood glucose; FFM, fat free mass; FM, fat mass; HDL-C, HDL cholesterol; HOMA-Index, homeostasis model assessment-index; LDL-C, LDL cholesterol; SBP, systolic blood pressure; WC, waist circumference; WHR, waist-to-hip ratio.

**Table 2:** Intra and intergroup changes of dietary intake data in the INT and CON group after 12 and 52 weeks of intervention (complete case analysis).

Complete Case Analysis	Baseline		12 Weeks		*p*	52 Weeks		*p*
	INT	CON	INT	CON	(INT vs. CON)	INT	CON	(INT vs. CON)
Energy (kcal)	2129 ± 580	2208 ± 587	−372 [−556; −174] ***	−288 [−625; 55] *	0.112	−217 [−440; 8.80] *	−493 [−809; −154] ***	0.389
Protein (g)	91 ± 21	92 ± 24	9.10 [1.05; 18.6] *	1.40 [−13.8; 14.9]	0.680	5.72 [−2.35; 13.2]	−16.4 [−26.0; −2.31] *	0.075
Protein (proportion of daily energy intake (%))	18.3 ± 5.5	17.4 ± 3.2	6.37 [4.69; 8.04] ***	2.48 [0.73; 4.23] **	<0.001	2.86 [1.40; 4.32] ***	1.45 [0.04; 2.86]	0.052
Fat (g)	90.1 ± 30.2	94.2 ± 29.2	−20.7 [−30.9; −10.1] ***	−9.72 [−28.3; 8.64]	0.051	−13.5 [−23.6; −3.02] **	−24.3 [−38.7; −6.91] **	0.509
Fat (proportion of daily energy intake (%))	40.0 ± 6.5	39.4 ± 6.1	−3.08 [−4.82; −1.34] ***	1.08 [−1.23; 3.39]	0.006	−2.11 [−3.87; −0.36] *	−0.65 [−3.21; 1.91]	0.424
Carbohydrates (g)	198 ± 71	215 ± 69	−52.0 [−67.2; −36.8] ***	−45.2 [−67.6; −22.7] ***	0.046	−28.2 [−46.3; −10.2] **	−54.6 [−78.7; −30.5] ***	0.416
Carbohydrates (proportion of daily energy intake (%))	38.9 ± 7.5	40.4 ± 7.4	−3.22 [−4.82; −1.63] ***	−3.27 [−5.52; −1.02] **	0.008	−1.24 [−3.06; 0.57]	−1.27 [−4.16; 1.63]	0.362
Glucose (g)	10.6 ± 6.6	12.8 ± 8.3	−3.23 [−4.54; −1.93] ***	−2.34 [−5.37; 0.68]	<0.001	−1.29 [−3.00; 0.42] *	−3.10 [−6.57; 0.37] *	0.981
Fructose (g)	13.0 ± 8.3	17.1 ± 12.8	−3.46 [−5.21; −1.71] ***	−3.51 [−8.15; 1.12]	<0.001	−0.92 [−3.21; 1.38]	−4.41 [−9.54; 0.72]	0.969
Sucrose (g)	36.2 ± 21.2	39.8 ± 21.0	−13.7 [−18.8; −8.60] ***	−11.9 [−19.5; −4.25] **	0.144	−9.58 [−15.2; −4.01] **	−15.2 [−22.5; −7.87] ***	0.814
Alcohol (g)	16.3 ± 17.1	15.3 ± 18.7	−2.38 [−6.74; 2.32]	−1.21 [−9.89; 7.11]	0.614	0.07 [−5.92; 6.02]	−0.82 [−8.91; 7.94]	0.663
Alcohol (proportion of daily energy intake (%))	4.62 ± 6.03	4.56 ± 5.75	−0.24 [−1.13; 0.65]	−0.11 [−1.60; 1.38]	0.884	0.29 [−0.98; 1.56]	0.28 [−1.24; 1.79]	0.861
Dietary fibre (g)	17.4 ± 7.4	20.2 ± 7.2	−2.37 [−3.69; −1.04] ***	−0.68 [−2.84; 1.47]	0.008	0.24 [−1.62; 2.10]	−2.60 [−4.77; −0.43] *	0.171
Weight (kg)	92.8 ± 10.1	92.2 ± 10.4	−7.23 [−8.37; −6.13] ***	−4.84 [−6.35; −3.22] ***	0.008	−5.27 [−6.82; −3.81] ***	−4.45 [−7.01; −1.91] *	0.469

Data are shown as mean [95% CI] or mean ± SD. Within-group changes after 12 and 52 weeks were analysed using Wilcoxon Test (in case of normal distribution using paired *t*-Test). *** *p* < 0.001 vs. baseline; ** *p* < 0.01 vs. baseline; * *p* < 0.05 vs. baseline. Differences in changes after 12 as well as 52 weeks between both groups were analyzed using ANCOVAs adjusting for baseline values and partly for the interaction term ‘group × baseline value’. Week 12: (INT: *n* = 82 [Men (*n* = 32) Women (*n* = 50)]), (CON: *n* = 37 [Men (*n* = 13) Women (*n* = 24)]); Week 52: (INT: *n* = 72 [Men (*n* = 29) Women (*n* = 43)], (CON: *n* = 32 [Men (*n* = 11) Women (*n* = 21)]).

## Data Availability

The data presented in this study are available on reasonable request from the corresponding author when Almased-Wellness-GmbH gave permission.

## Supplementary Materials

The following are available online at https://www.mdpi.com/2072-6643/13/2/376/s1, Table S1: Baseline characteristics for ACOORH completers and participants with correctly filled dietary records after 12 weeks, Table S2: Intra and intergroup changes of dietary intake data in the INT and CON group after 12 and 52 weeks of intervention stratified by sex (complete case analysis).
