# Supplementary material for: Effects of a Protein-Rich, Low-Glycaemic Meal Replacement on Changes in Dietary Intake and Body Weight Following a Weight-Management Intervention—The ACOORH Trial
Source: Nutrients. 2021 Jan 26;13(2):376. doi: 10.3390/nu13020376 (PMC7910938; doi:10.3390/nu13020376)
Supplement: Supplementary file 1 [file nutrients-13-00376-s001.pdf]

nutrients

MDPI

Supplementary materials Table S1. Baseline characteristics for ACOORH completers and participants with correctly filled dietary records after 12 weeks

	Completers (n=317)	Participants with correctly filled out diet diaries (n=119)
Sex (% male)	36.9	37.8
Age (years)	51 ± 9	52 ± 8
Weight (kg)	92 ± 13	92 ± 13
BMI (kg/m^2)	31.5 ± 2.3	31.3 ± 2.4
WC (cm)	106 ± 9	105 ± 9
WHR	0.95 ± 0.08	0.95 ± 0.08
FM (kg)	36.7 ± 6.5	36.5 ± 6.4
FFM (kg)	55.6 ± 11.7	55.7 ± 11.3
HbA_{1c} (%)	5.50 ± 0.35	5.48 ± 0.32
FBG (mg/dl)	94 ± 12	92 ± 10
SBP (mmHg)	134 ± 15	135 ± 15
DBP (mmHg)	89 ± 12	91 ± 11
Total cholesterol (mg/dl)	218 ± 40	215 ± 41
HDL-C (mg/dl)	56 ± 15	54 ± 13
LDL-C (mg/dl)	138 ± 35	137 ± 35
Triglycerides (mg/dl)	143 ± 74	147 ± 74

Shown are means ± standard deviations, or percentages. BMI, body mass index; DBP, diastolic blood pressure; FBI, fasting blood insulin; FBG, fasting blood glucose; FM, fat mass; FFM, fat free mass; HDL- C, HDL cholesterol; HOMA-Index, homeostasis model assessment-index; LDL-C, LDL cholesterol; SBP, systolic blood pressure; WC, waist circumference; WHR, waist-to-hip ratio

Supplementary materials Table S2. Intra and intergroup changes of dietary intake data in the INT and CON group after 12 and 52 weeks of intervention stratified by sex (complete case analysis)

Complete case analysis		12 weeks			52 weeks		
	Sex	INT (n≈82) [Men (n≈32) Women (n≈50)]	CON (n≈37) [Men (n≈13) Women (n≈24)]	P (INT vs. CON)	INT (n≈72) [Men (n≈29) Women (n≈43)]	CON (n≈32) [Men (n≈11) Women (n≈21)]	P (INT vs. CON)
Energy (kcal)	Male	-383 [-601; -165]**	-291 [-685; 103]	0.151	-208 [-471; 55.6]	-495 [-965; -24.4]*	0.512
Energy (kcal)	Female	-364 [-536; -191]***	-285 [-555; -14.7]*	0.078	-227 [-415; -38.8]*	-491 [-669; -314]***	0.169
Protein (g)	Male	6.50 [-4.66; 17.65]	7.12 [-10.1; 24.3]	0.967	0.24 [-12.0; 12.4]	-16.3 [-35.3; 2.68]	0.149
Protein (g)	Female	12.7 [6.18; 19.3]***	-5.05 [-16.8; 6.68]	0.021	5.72 [-2.35; 13.8]	-16.4 [-26.0; -6.75]**	0.002
Fat (g)	Male	-18.6 [-30.3; -6.87]**	-8.83 [-29.5; 11.8]	0.070	-13.1 [-24.7; -1.38]*	-27.4 [-49.5; -5.27]*	0.528
Fat (g)	Female	-22.1 [-31.3; -12.9]***	-10.5 [-27.3; 6.24]	0.025	-13.8 [-22.9; -4.62]**	-19.6 [-31.4; -7.77]**	0.493
Alcohol (g)	Male	-1.57 [-7.65; 4.51]	-2.04 [-14.0; 9.93]	0.820	0.44 [-8.70; 9.59]	-0.07 [-11.3; 11.2]	0.864
Alcohol (g)	Female	-2.92 [-5.77; -0.07]*	-0.59 [-5.69; 4.51]	0.381	-0.28 [-3.52; 2.96]	-1.31 [-6.99; 4.36]	0.449

Data are shown as mean [95% CI]. Within-group changes after 12 and 52 weeks were analysed using Wilcoxon Test (in case of normal distribution using paired T-Test). *** $p < 0.001$ vs. baseline; ** $p < 0.01$ vs. baseline; * $p < 0.05$ vs. baseline. Differences in changes after 12 as well as 52 weeks between both groups were analyzed using ANCOVAs adjusting for baseline values and partly for the interaction term 'group x baseline value'.
